# Modified Plug Repair with Limited Sphincter Sparing Fistulectomy in the Treatment of Complex Anal Fistulas

**DOI:** 10.3389/fsurg.2014.00017

**Published:** 2014-05-30

**Authors:** Ferdinand Köckerling, Thomas von Rosen, Dietmar Jacob

**Affiliations:** ^1^Department of Surgery, Center of Minimally Invasive Surgery, Vivantes Hospital, Academic Teaching Hospital of Charité Medical School, Berlin, Germany

**Keywords:** anal fistula, plug repair, sphincter sparing fistulectomy, fistula plug, biological plug

## Abstract

**Purpose:** New technical approaches involving biologically derived products have been used to treat complex anal fistulas in order to avoid the risk of fecal incontinence. The least invasive methods involve filling out the fistula tract with fibrin glue or introduction of an anal fistula plug into the fistula canal following thorough curettage. A review shows that the new techniques involving biologically derived products do not confer any significant advantages. Therefore, the question inevitably arises as to whether the combination of a partial or limited fistulectomy, i.e., of the extrasphincteric portion of the fistula, and preservation of the sphincter muscle by repairing the section of the complex anal fistula running through the sphincter muscle and filling it with a fistula plug produces better results.

**Methods:** A modified plug technique was used, in which the extrasphincteric portion of the complex anal fistula was removed by means of a limited fistulectomy and the remaining section of the fistula in the sphincter muscle was repaired using the fistula plug with fixing button.

**Results:** Of the 52 patients with a complex anal fistula, who had undergone surgery using a modified plug repair with limited fistulectomy of the extrasphincteric part of the fistula and use of the fistula plug with fixing button, there are from 40 patients (follow-up rate: 77%) some kind of follow-up informations, after a mean of 19.32 ± 6.9 months. Thirty-two were men and eight were women, with a mean age of 52.97 ± 12.22 years. Surgery was conducted to treat 36 transsphincteric, 1 intersphincteric, and 3 rectovaginal fistulas. In 36 of 40 patients (90%), the complex anal fistulas or rectovaginal fistulas were completely healed without any sign of recurrence. None of these patients complained about continence problems.

**Conclusion:** A modification of the plug repair of complex anal fistulas with limited fistulectomy of the extrasphincteric part of the fistula and use of the plug with fixing button seems to increase the healing rate in comparison to the standard plug technique.

## Introduction

Recurrence and gas and/or stool incontinence are the most common complications after surgical treatment of fistula-in-ano (5–34 and 0–63%, respectively) (1). Therefore, the goals of the treatment of fistula-in-ano include resolving the acute-on-chronic inflammatory process, maintaining continence, and preventing future recurrence (2). Although laying open of the fistula tract has stood the test of time for the majority of low fistulas, it is a quite different story as the fistula becomes more complex (3). Complex fistulas, or those where a fistulotomy would result in incontinence, account for approximately 50% of cases with this disease process (2), including high transsphincteric, suprasphincteric, extrasphincteric, all anterior transsphincteric fistulas in women, and those caused by Crohn’s disease. This has led to a search for alternative sphincter-preserving techniques such as endorectal or dermal advancement flaps, prolonged seton drainage, fibrin sealant injection, biological or bioprosthetic materials, and most recently the potential utility of stem cells (3).

The promising results reported by some authors regarding the two least invasive conservative methods, fibrin glue and Surgisis AFP™ anal fistula plug, are interesting; however, their efficacy in healing the fistulas needs to be better evaluated. Healing rates from 31 to 85% have been reported for fibrin glue and from 14 to 87% for the plug (4) Since some authors reported only somewhat moderate success of 50–60% with the use of the biological anal fistula plug in their studies (5), they pointed out that further modifications of the plug technique or utilization of other biological materials will most likely play an additional role in the treatment of anal fistulas in the future (3).

In the studies on which the aforementioned findings are based, the plug technique was carried out in the following way: once all inflammatory disease had resolved over a period of 6–8 weeks, the plug was drawn snug at the internal opening and sutured in place, and then cut flush at the external opening without fixation at this location (2). As such, when applying this technique up till now, implementation of a fistulectomy or of a total or partial/limited fistulectomy was fully omitted. Now there are a number of publications reporting on relatively good results for treatment of complex anal fistulas through sphincter-preserving total or limited/partial fistulectomy in combination with fibrin glue or suture closure of the internal opening of the anal fistula (6–8).

Here, we present the results of a modified plug technique in which the extrasphincteric portion of the complex anal fistula was removed by means of a limited fistulectomy and the remaining section of the fistula in the sphincter muscle was repaired using the fistula plug with fixing button.

## Patients and Methods

### Patients

The study group comprised 52 consecutive patients, who underwent surgery in our hospital between 15 January 2009 and 31 December 2012 because of a complex anal fistula (Table 1). Of the 52 patients, it was possible to obtain follow-up data for 40 after a mean of 19.32 ± 6.97 months (follow-up rate: 77%). Of the 40 patients, 28 underwent clinical and rectoscopic reexamination. Twelve patients were interviewed in detail by telephone about any persistent symptoms or further treatments.

**Table 1:** **Characteristics of patients with follow-up (*n* = 40)**.

Male	*n* = 32 (80%)
Female	*n* = 8 (20%)
Mean age	52.97 ± 12.22 years
Transsphincteric fistula	*n* = 36 (90%)
Intersphincteric fistula	*n* = 1 (2.5%)
Rectovaginal fistula	*n* = 3 (7.5%)
Mean follow-up	19.32 ± 6.97 months

Of the patients for whom follow-up data were available, 32 were men and 8 were women, with a mean age of 52.97 ± 12.22 years. These patients were treated by four surgeons. Here, surgery was conducted to treat 36 transsphincteric, 1 intersphincteric, and 3 rectovaginal fistulas. For 14 patients, the complex anal fistula was surgically treated concomitantly with opening of a periproctitic abscess. Hemorrhoids were concurrently removed in two patients. For four patients, surgery was conducted because of a recurrent fistula and two patients suffered from Crohn’s disease.

### Surgical technique

**Figure 1:**
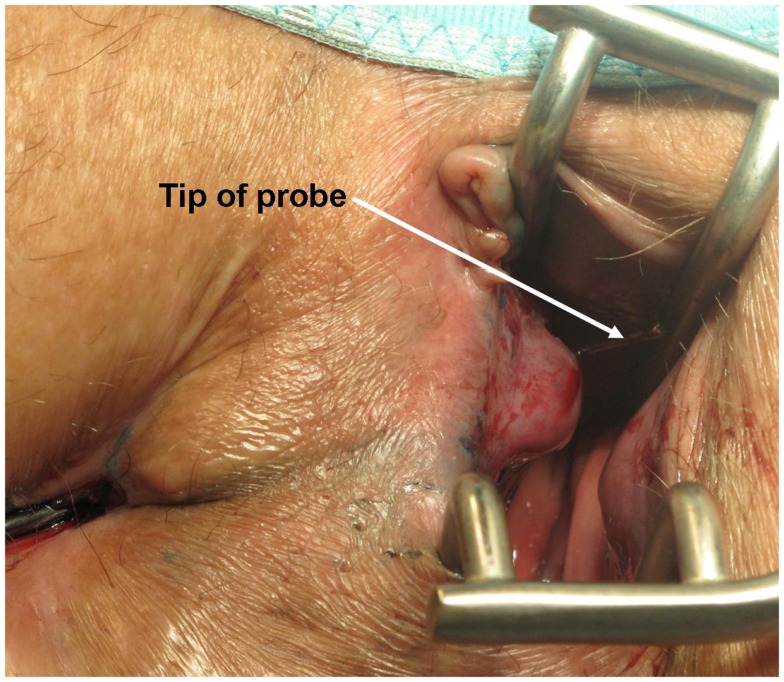
**Marking of the complex anal fistula with a flexible metallic probe**.

**Figure 2:**
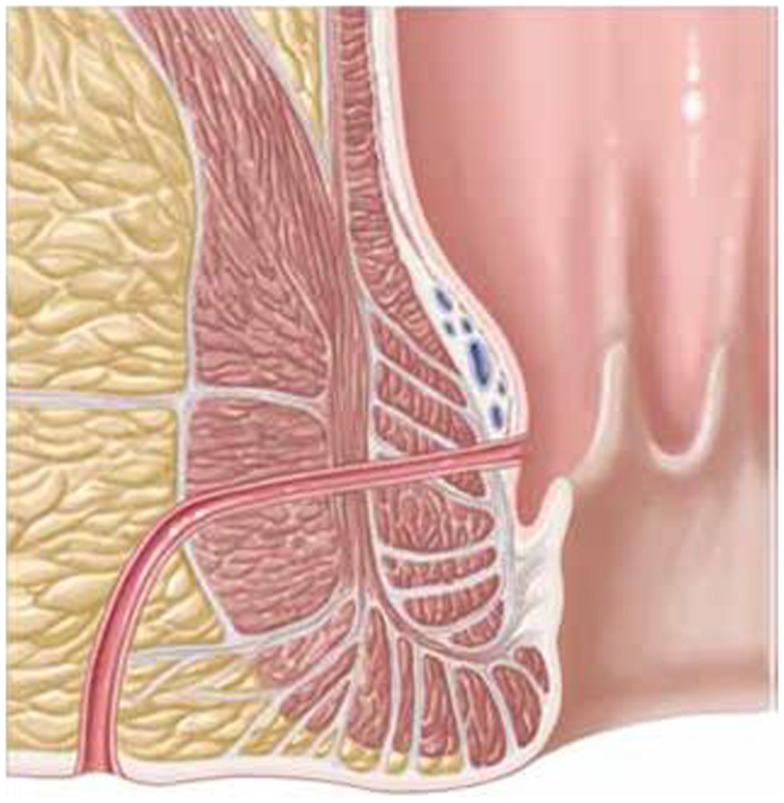
**Schematic drawing of a transsphincteric anal fistula**.

**Figure 3:**
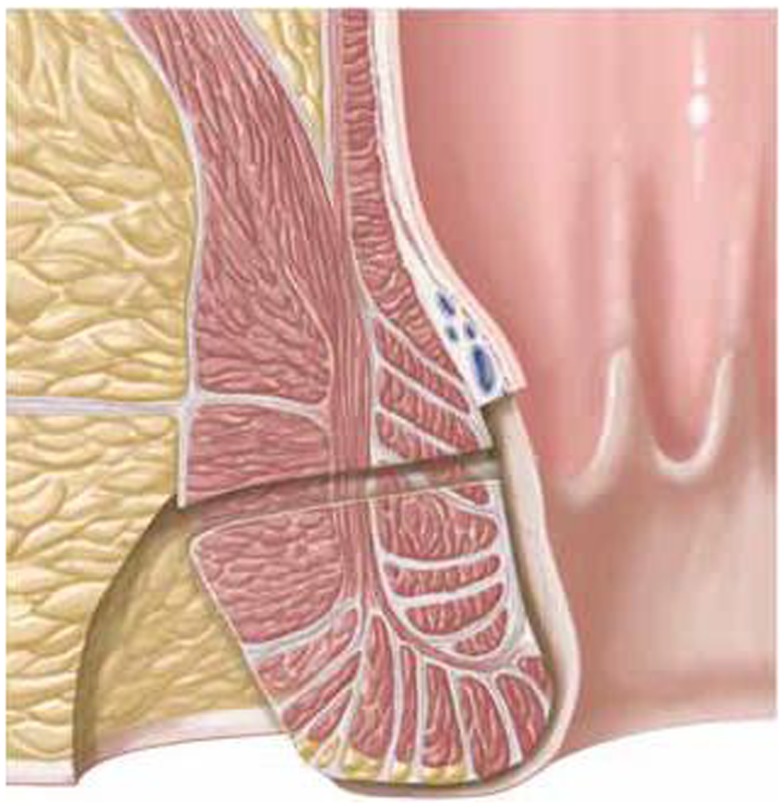
**Limited or partial fistulectomy with preservation of the sphincter muscle**. The external opening, the extrasphincteric part of the fistula, and the internal opening are removed.

**Figure 4:**
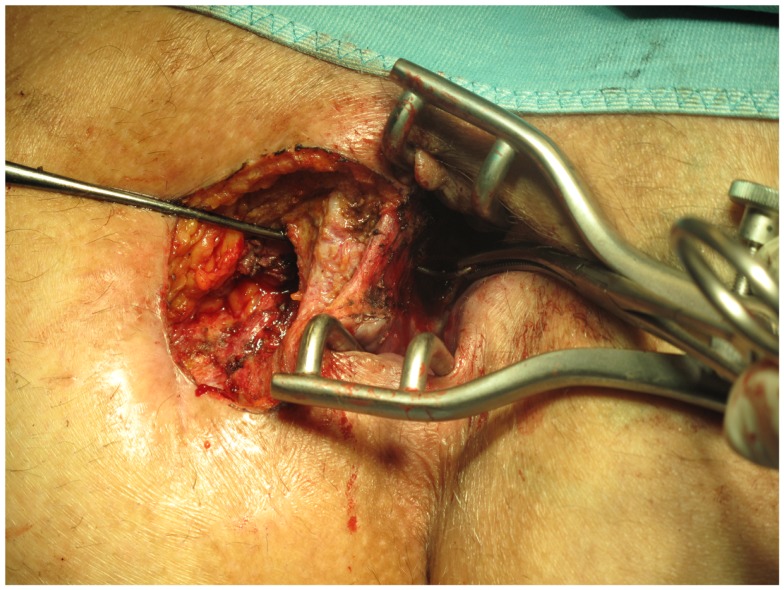
**Clinical picture after limited or partial removal of the complex anal fistula and preservation of the internal and external sphincter muscle**.

**Figure 5:**
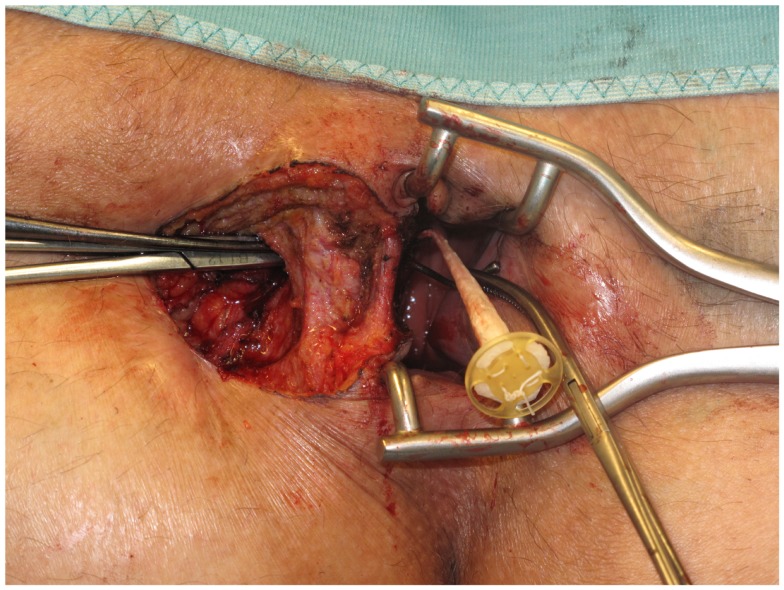
**Introduction of the anal fistula plug with fixing button into the remaining intrasphincteric part of the complex anal fistula**.

**Figure 6:**
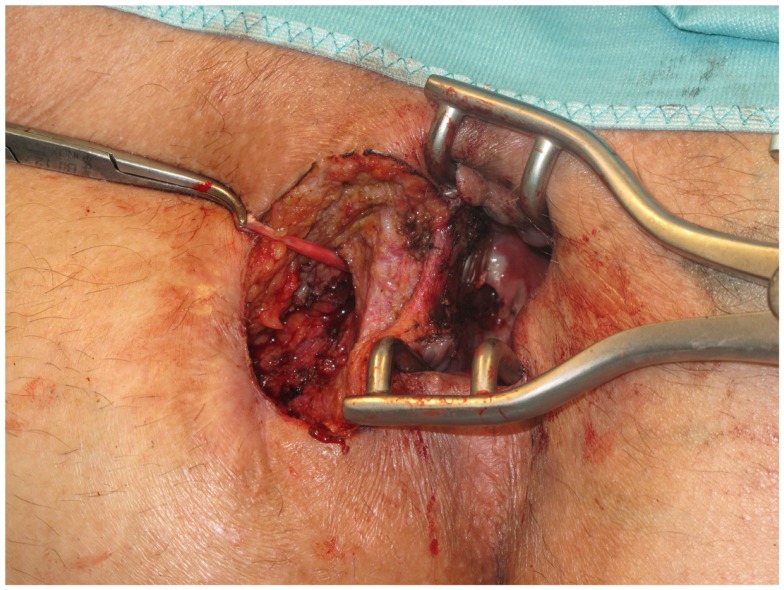
**For tight securing of the fixing button to the sphincter muscle, the plug is pulled by a forceps into the remaining intrasphincteric part of the complex anal fistula from outside**.

**Figure 7:**
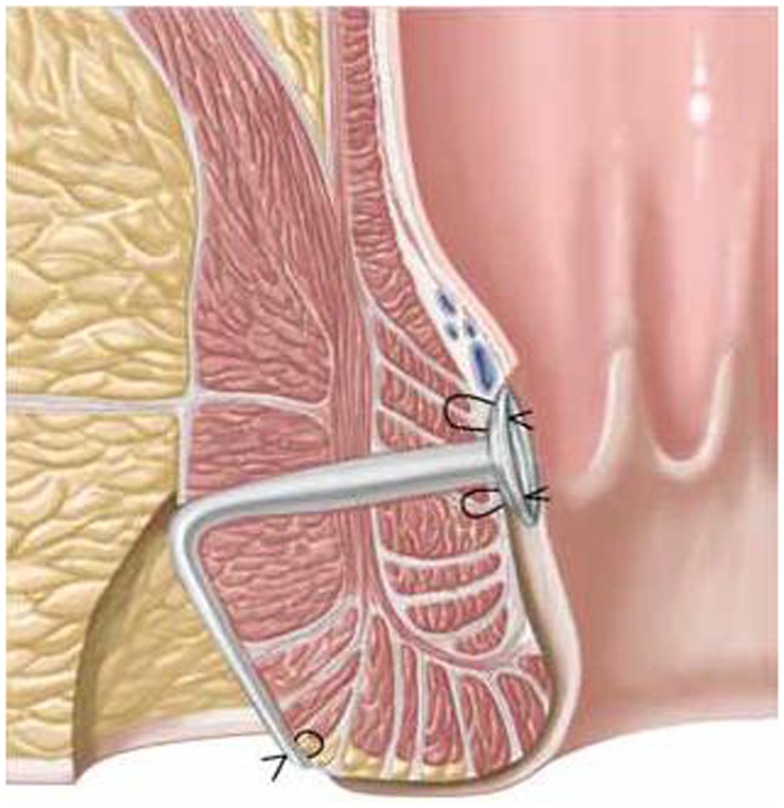
**Fixation of the fixing button internally with several absorbable single button sutures to the underlying sphincter muscle**.

**Figure 8:**
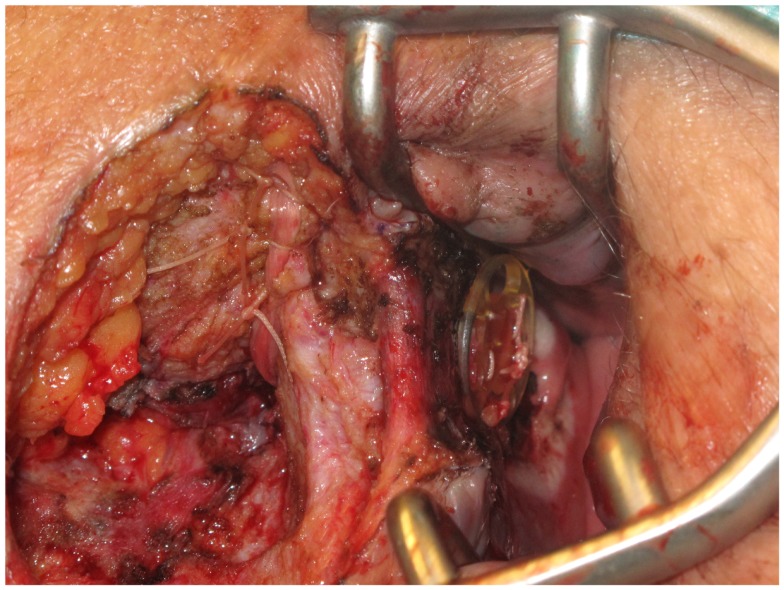
**The external end of the fistula plug is also secured with several absorbable single button sutures to the base of the wound of the limited fistulectomy**.

First, the internal fistula opening was exposed using an anal retractor and the entire fistula tract was marked with a flexible metallic probe (Figure 1). The metallic probe was secured internally with a clamp to prevent it from becoming displaced. Next, limited or partial fistulectomy was conducted while preserving the sphincter muscle (Figures 2 and 3). In doing so, starting from the outside the extrasphincteric portion of the complex anal fistula was cut out using diathermy, while preserving the sphincter muscle. The sphincter muscle was then exposed, also working from the outside inwards as far as the internal fistula opening (Figures 3 and 4). As such, both the internal and external opening of the complex anal fistula as well as the extrasphincteric portion of the fistula was removed, leaving behind only the section of the complex anal fistula located within the sphincter muscle. Next, a fistula plug with fixing button was introduced into this remaining portion of the complex anal fistula (Surgisis AFP™ fistula plug with fixing button, now called Biodesign™ fistula plug with fixing button) (Figure 5), while securing tightly to the sphincter muscle the fixing button of the plug by pulling the end of the plug (Figure 6). The fixing button was fixed internally with several absorbable single button sutures to the underlying sphincter muscle such that the remaining internal opening of the fistula was closed as tightly as possible (Figure 7). Then the external end of the fistula plug was also secured with several absorbable single button sutures to the base of the wound of the limited fistulectomy, while pulling tightly (Figures 7 and 8). Thanks to the voluminous fistula plug with button, the remaining fistula portion in the sphincter muscle was filled out well and generally well-sealed. On completion of the operation, the remaining fistula portion in the sphincter muscle should be rendered virtually “water tight” by the fistula plug with fixing button. Post-surgical treatment of patients was similar to that practiced following other proctologic procedures with thrice daily irrigation of the open wound.

## Results

Of the 52 patients with complex anal fistula who had undergone surgery using the technique described above, 40 presented for follow-up examination after a mean of 19.32 ± 6.97 months (77%). Twenty-eight patients underwent clinical and rectoscopic reexamination. Twelve patients were interviewed in detail on the telephone, who, in the absence of symptoms, were not willing to undergo clinical and rectoscopic reexamination. A persistent transsphincteric anal fistula was detected in three patients, with Crohn’s disease being implicated as the cause of the anal fistula in one patient. In another patient, there was initial closure of the transsphincteric portion of the anal fistula, but the extrasphincteric wound did not heal after limited fistulectomy. Renewed intervention then revealed that this was due to the presence of a further transsphincteric anal fistula, which was then also treated by means of a plug. But even after this subsequent fistula operation, the extrasphincteric wound healed only very slowly. In 36 of the remaining 40 patients (90%), the complex anal fistulas or rectovaginal fistulas described here healed completely without any sign of recurrence after a mean of 19.32 ± 6.97 months. None of these patients complained about continence problems.

## Discussion

New technical approaches involving biologically derived products have been used to treat complex anal fistulas in order to avoid the risk of fecal incontinence (9). The least invasive methods involve filling out the fistula tract with fibrin glue or introduction of a Surgisis AFP™ anal fistula plug into the fistula canal following thorough curettage. The cure rates given in the literature for these least invasive techniques are 31–85% for fibrin glue and 14–87% for the fistula plug (4). A review shows that the new techniques involving biologically derived products do not confer any significant advantages (9). Another review found an overall mean clinical success of 50–60% (5). Hence, the technique described hitherto for treatment of complex anal fistulas with the fistula plug, without invasive treatment of the fistula, does not appear to produce sufficiently reliable results with regard to healing of a complex anal fistula. Therefore, the question inevitably arises as to whether the combination of a partial or limited fistulectomy, i.e., of the extrasphincteric portion of the fistula, and preservation of the sphincter muscle by repairing the section of the complex anal fistula running through the sphincter muscle and filling it with a fistula plug produces better results. This technical procedure is also supported by publications, reporting on relatively good results for complex anal fistulas by means of partial or limited or total fistulectomy in combination with fibrin glue or suture closure of the internal opening (6–8). Total fistulectomy with simple closure of the internal opening can be effective for the long-term closure of complex cryptoglandular fistulas (6, 7). Alternatively, the fistula track is partially existed and followed by instillation of fibrin glue (8). The technique described here has a similar aim: excision of the fistula portions outside the sphincter muscle, closure of the internal fistula opening in the sphincter muscle using the fistula plug with button, and filling the section of the complex anal fistula running through the sphincter muscle with a biologically derived product. A cure rate of 90% without any adverse effect on continence is very promising for complex anal fistulas. But, of course, the present study has limitations. The number of cases is relatively small. It was not possible to carry out clinical and rectoscopic reexamination for all patients. To evaluate realistically the significance of this technique, larger multicenter, prospective randomized trials must now be conducted. But the existing data demonstrate that the results obtained for plug therapy of complex, transsphincteric anal fistulas can be improved by using a modified technique involving partial or limited fistulectomy. This means that the method will be naturally more invasive compared to the technique used hitherto. But this modified procedure appears more suited to reaching the goal of bringing about healing of a complex anal fistula by means of a single operative procedure, while preserving the sphincter muscle.

## Conflict of Interest Statement

The authors declare that the research was conducted in the absence of any commercial or financial relationships that could be construed as a potential conflict of interest.

## References

[1] Sygut A Mik M Trzeinski R Dziki A. How the location of the internal opening of anal fistulas affect the treatment results of primary transsphincteric fistulas? Langenbecks Arch Surg (2010) 395:1055–9. 10.1007/s00423-009-0562-0 19924437

[2] Dukukgian H Abcarian H. Why do we have so much trouble treating anal fistula? World J Gastroenterol (2011) 17:3292–6. 10.3748/wjg.v17.i28.3292 21876616 PMC3160532

[3] Cintron JR Abcarian H Chaudhry V Singer M Hunt S Birnbaum E Treatment of fistula-in-ano using a porcine small intestinal submucosa anal fistula plug. Tech Coloproctol (2013) 17:187–91. 10.1007/s10151-012-0897-3 23053440

[4] Fucini C Giani I. Why do we have to review our experience in managing cases with idiopathic fistula-in-ano regularly? World J Gastroenterol (2011) 17:3297–9. 10.3748/wjg.v17.i28.3297 21876617 PMC3160533

[5] Lewis R Lunniss PJ Hammond TM. Novel biological strategies in the management of anal fistula. Colorectal Dis (2012) 14:1445–56. 10.1111/j.1463-1318.2012.03192.x 22882376

[6] Tobisch A Stelzner S Hellmich G Jackisch T Witzigmann H. Total fistulectomy with simple closure of the internal opening in the management of complex cryptoglandular fistulas: long-term results and functional outcome. Dis Colon Rectum (2012) 55:750–5. 10.1097/DCR.0b013e3182569b29 22706126

[7] Mangual RN Tudu DN Pattnaik SP Mohanty SS Prusty KP The sphincter preserving perianal fistulectomy: a better alternative. Indian J Surg (2004) 66:31–5.

[8] Hassan MZM Nor AM Limited fistulectomy and fibrin glue for the treatment of complex fistula-in-ano. World J Colorectal Surg (2008) 1:8.

[9] Cirocchi R Trastulli S Morelli U Desiderio J Boselli C Parisi A The treatment of anal fistulas with biologically derived products: is innovation better than conventional surgical treatment? Tech Coloproctol (2013) 17:259–73. 10.1007/s10151-012-0948-9 23207714

